# Supplementary material for: Strong functional data for pathogenicity or neutrality classify BRCA2 DNA-binding-domain variants of uncertain significance
Source: Am J Hum Genet. 2021 Feb 19;108(3):458–68. doi: 10.1016/j.ajhg.2021.02.005 (PMC8008494; doi:10.1016/j.ajhg.2021.02.005)
Supplement: Document S1. Supplemental references [file mmc1.pdf]

The American Journal of Human Genetics, Volume *108*

Supplemental data

Strong functional data for pathogenicity or neutrality classify BRCA2 DNA-binding-domain variants of uncertain significance

Marcy E. Richardson, Chunling Hu, Kun Y. Lee, Holly LaDuca, Kelly Fulk, Kate M. Durda, Ashley M. Deckman, David E. Goldgar, Alvaro N.A. Monteiro, Rohan Gnanaolivu, Steven N. Hart, Eric C. Polley, Elizabeth Chao, Tina Pesaran, and Fergus J. Couch

Supplemental Data

Table S1- HDR functional assay results for all 252 variants. Results from other BRCA2 functional studies, multifactorial analyses, and splice predictions are shown to allow for comparison with HDR data.

Supplemental references

1. Guidugli, L., Pankratz, V.S., Singh, N., Thompson, J., Erding, C.A., Engel, C., Schmutzler, R., Domchek, S., Nathanson, K., Radice, P., et al. (2013). A classification model for BRCA2 DNA binding domain missense variants based on homology-directed repair activity. Cancer Res 73, 265-275.

2. Guidugli, L., Shimelis, H., Masica, D.L., Pankratz, V.S., Lipton, G.B., Singh, N., Hu, C., Monteiro, A.N.A., Lindor, N.M., Goldgar, D.E., et al. (2018). Assessment of the Clinical Relevance of BRCA2 Missense Variants by Functional and Computational Approaches. Am J Hum Genet 102, 233-248.

3. Hart, S.N., Hoskin, T., Shimelis, H., Moore, R.M., Feng, B., Thomas, A., Lindor, N.M., Polley, E.C., Goldgar, D.E., Iversen, E., et al. (2019). Comprehensive annotation of BRCA1 and BRCA2 missense variants by functionally validated sequence-based computational prediction models. Genetics in medicine : official journal of the American College of Medical Genetics 21, 71-80.

4. Mesman, R.L.S., Calleja, F., Hendriks, G., Morolli, B., Misovic, B., Devilee, P., van Asperen, C.J., Vrieling, H., and Vreeswijk, M.P.G. (2019). The functional impact of variants of uncertain significance in BRCA2. Genetics in medicine : official journal of the American College of Medical Genetics 21, 293-302.

5. Biswas, K., Das, R., Eggington, J.M., Qiao, H., North, S.L., Stauffer, S., Burkett, S.S., Martin, B.K., Southon, E., Sizemore, S.C., et al. (2012). Functional evaluation of BRCA2 variants mapping to

the PALB2-binding and C-terminal DNA-binding domains using a mouse ES cell-based assay. Hum Mol Genet 21, 3993-4006.

6. Kuznetsov, S.G., Liu, P., and Sharan, S.K. (2008). Mouse embryonic stem cell-based functional assay to evaluate mutations in BRCA2. Nat Med 14, 875-881.

7. Ikegami, M., Kohsaka, S., Ueno, T., Momozawa, Y., Inoue, S., Tamura, K., Shimomura, A., Hosoya, N., Kobayashi, H., Tanaka, S., et al. (2020). High-throughput functional evaluation of BRCA2 variants of unknown significance. Nat Commun 11, 2573.

8. Alter, B.P., Rosenberg, P.S., and Brody, L.C. (2007). Clinical and molecular features associated with biallelic mutations in FANCD1/BRCA2. J Med Genet 44, 1-9.

9. Dodgshun, A.J., Sexton-Oates, A., Saffery, R., and Sullivan, M.J. (2016). Biallelic FANCD1/BRCA2 mutations predisposing to glioblastoma multiforme with multiple oncogenic amplifications. Cancer Genet 209, 53-56.

10. Hirsch, B., Shimamura, A., Moreau, L., Baldinger, S., Hag-alshiekh, M., Bostrom, B., Sencer, S., and D'Andrea, A.D. (2004). Association of biallelic BRCA2/FANCD1 mutations with spontaneous chromosomal instability and solid tumors of childhood. Blood 103, 2554-2559.

11. Tian, Y., Pesaran, T., Chamberlin, A., Fenwick, R.B., Li, S., Gau, C.L., Chao, E.C., Lu, H.M., Black, M.H., and Qian, D. (2019). REVEL and BayesDel outperform other in silico meta-predictors for clinical variant classification. Sci Rep 9, 12752.

12. Lindor, N.M., Guidugli, L., Wang, X., Vallee, M.P., Monteiro, A.N., Tavtigian, S., Goldgar, D.E., and Couch, F.J. (2012). A review of a multifactorial probability-based model for classification of BRCA1 and BRCA2 variants of uncertain significance (VUS). Hum Mutat 33, 8-21.

13. Easton, D.F., Deffenbaugh, A.M., Pruss, D., Frye, C., Wenstrup, R.J., Allen-Brady, K., Tavtigian, S.V., Monteiro, A.N., Iversen, E.S., Couch, F.J., et al. (2007). A systematic genetic assessment of 1,433

sequence variants of unknown clinical significance in the BRCA1 and BRCA2 breast cancer-predisposition genes. Am J Hum Genet 81, 873-883.

14. Parsons, M.T., Tudini, E., Li, H., Hahnen, E., Wappenschmidt, B., Feliubadalo, L., Aalfs, C.M., Agata, S., Aittomaki, K., Alducci, E., et al. (2019). Large scale multifactorial likelihood quantitative analysis of BRCA1 and BRCA2 variants: An ENIGMA resource to support clinical variant classification. Hum Mutat 40, 1557-1578.
