# Supplementary material for: Strong functional data for pathogenicity or neutrality classify BRCA2 DNA-binding-domain variants of uncertain significance
Source: Am J Hum Genet. 2021 Feb 19;108(3):458–68. doi: 10.1016/j.ajhg.2021.02.005 (PMC8008494; doi:10.1016/j.ajhg.2021.02.005)
Supplement: Document S2. Article plus supplemental information [file mmc3.pdf]

ARTICLE

Strong functional data for pathogenicity or neutrality classify BRCA2 DNA-binding-domain variants of uncertain significance

Marcy E. Richardson,[1] Chunling Hu,[2] Kun Y. Lee,[2] Holly LaDuca,[1] Kelly Fulk,[1] Kate M. Durda,[1] Ashley M. Deckman,[1] David E. Goldgar,[3] Alvaro N.A. Monteiro,[4] Rohan Gnanaolivu,[2] Steven N. Hart,[2] Eric C. Polley,[2] Elizabeth Chao,[1] Tina Pesaran,[1] and Fergus J. Couch[2,*]

Summary

Determination of the clinical relevance of rare germline variants of uncertain significance (VUSs) in the *BRCA2* cancer predisposition gene remains a challenge as a result of limited availability of data for use in classification models. However, laboratory-based functional data derived from validated functional assays of known sensitivity and specificity may influence the interpretation of VUSs. We evaluated 252 missense VUSs from the BRCA2 DNA-binding domain by using a homology-directed DNA repair (HDR) assay and identified 90 as non-functional and 162 as functional. The functional assay results were integrated with other available data sources into an ACMG/AMP rules-based classification framework used by a hereditary cancer testing laboratory. Of the 186 missense variants observed by the testing laboratory, 154 were classified as VUSs without functional data. However, after applying protein functional data, 86% (132/154) of the VUSs were reclassified as either likely pathogenic/pathogenic (39/132) or likely benign/benign (93/132), which impacted testing results for 1,900 individuals. These results indicate that validated functional assay data can have a substantial impact on VUS classification and associated clinical management for many individuals with inherited alterations in *BRCA2*.

Introduction

Germline pathogenic variants (PVs) in *BRCA2* are associated with a significantly increased risk of breast, ovarian, pancreatic, and prostate cancers (MIM: 600185).[1] The detection and accurate classification of PVs in *BRCA2*, among other hereditary cancer genes, proffers a notable benefit to carriers of disease-causing variants and their biological family members found to carry the same PVs who can benefit from enhanced cancer surveillance for the early detection of hereditary cancer and/or pursue prophylactic measures to aid in the prevention of certain cancers. In addition, carriers of PVs with cancer may become eligible and benefit from polyADP-ribose-polymerase (PARP)-inhibitor treatment for advanced/recurrent ovarian cancer and metastatic breast, pancreatic, and prostate cancers (see "NCCN clinical practice guidelines in oncology" in web resources).[2,3] However, through the widespread adoption of multi-gene panel testing, the detection of rare *BRCA2* variants has outpaced the ability to assess the clinical relevance of the variants, leading to a disproportionate number of variants of uncertain significance (VUSs). Furthermore, independent data and variation in interpretations of classification criteria by genetic testing facilities has led to discordance in variant classification in the public domain.[4] Such VUSs and variants with conflicting interpretation pose a challenge to clinical counseling and management of patients and biological relatives.[5]

In an attempt to harmonize classification criteria, the American College of Medical Genetics and Genomics/Association for Molecular Pathology (ACMG/AMP) developed variant classification standards and guidelines that provided a five-tiered variant classification framework.[6] Although this framework provides a robust starting point for most genes, classification of rare variants remains challenging as a result of limited availability of phenotype and genotype information. However, the guidelines must be tailored for gene-specific considerations because not all rules can be applied uniformly. For instance, interpretation of rare variants in the *BRCA1* and *BRCA2* cancer predisposition genes is complicated by the limited applicability of phenotype-driven rules that involve assessment of the presence or absence of breast and ovarian cancer given the relatively high frequency of these phenotypes in the general population. Similarly, ACMG/AMP framework rules based on functional assay data are often not effectively used for ACMG/AMP rules-based variant classification because of the absence of validated assays with established sensitivity and specificity for pathogenic variants.[7] Classification of missense VUSs in commonly mutated genes, such as *BRCA1* and *BRCA2*, may benefit from development of high-quality functional assays that have been calibrated relative to established pathogenic and benign missense variants. In this study, we characterized missense variants in the BRCA2 DNA-binding domain (DBD) by using a homology-directed DNA repair (HDR) assay with high sensitivity and specificity.[8–10] We subsequently combined the results with data elements

[1]Ambry Genetics, Aliso Viejo, CA 92656, USA; [2]Mayo Clinic, Rochester, MN 55905, USA; [3]University of Utah, Salt Lake City, UT 84132, USA; [4]Moffitt Cancer Center, Tampa, FL 33612, USA
*Correspondence: couch.fergus@mayo.edu
https://doi.org/10.1016/j.ajhg.2021.02.005.
© 2021 The Authors. This is an open access article under the CC BY license (http://creativecommons.org/licenses/by/4.0/).

from a clinical diagnostic laboratory (Ambry Genetics) to classify BRCA2 DBD VUSs by using a tailored ACMG/AMP variant classification framework.[6,11,12]

Material and methods

Variant selection

Missense variants (n = 252) in the BRCA2 DBD (amino acids 2481–3186) were selected for functional analysis based on observation in ClinVar.[4] These included 20 missense variants established as likely pathogenic/pathogenic (LP/P) and 46 variants established as likely benign/benign (LB/B) via models that were independent of functional data.[11,13–16] Additional VUSs reported in ClinVar were selected on the basis of BRCA-ML *in silico* sequence-based prediction model scores (>0.551), which are associated with an odds of pathogenicity (sensitivity/[1-specificity]) > 3.0 (data not shown).[10] Variants predicted by SpliceAI[17] or known from other studies to have effects on RNA splicing were intentionally excluded from this dataset such that the remaining variants were exclusively evaluated for effects on protein function. Variants are presented according to HGVS recommendations and correspond to the RefSeq transcript ID GenBank: NM_000059.3.

Homology-directed repair (HDR) assay

The BRCA2 homology-directed DNA repair (HDR) assay has been described previously.[9] In brief, variants were incorporated by site-directed mutagenesis into a mammalian expression construct containing the *BRCA2* coding sequence (GenBank: NM_000059.3) that encodes a 3× FLAG-tagged full-length BRCA2 protein (GenBank: NP_000050.2). The presence of variants was verified by plasmid sequencing. *BRCA2* expression constructs and the iSce1 expression plasmid were co-transfected into DR-GFP *brca2*-deficient V-C8 cells.[8–10,14,18,19] All variants were analyzed in duplicate experiments for at least two independently derived clones. BRCA2 expression was verified by immunoblot. For each transfection, the proportion of viable cells displaying GFP expression (GFP+) was quantified by flow cytometry. Fold increases in GFP+ cells, which are equivalent to HDR-fold changes, were normalized and rescaled to a 1:5 ratio derived from the p.Asp2723His (c.8167G>C) pathogenic variant control and the benign wild-type BRCA2 control. We used a Bayesian regression model for the log HDR scores to estimate the distribution of HDR scores for all variants.[9,10] The posterior distribution for the HDR score and the 2.5 and 97.5 percentiles of the posterior distributions were used to assign 95% confidence intervals for all variants. All estimation was done in R with the rstanarm package.[20] The sensitivity and specificity of the HDR assay for pathogenic missense variants in the DBD of BRCA2 has previously been estimated at 100% (sensitivity, 95% CI: 79%–100%; specificity, 95% CI: 93%–100%) with known neutral and known pathogenic variants.[9]

ACMG/AMP rule-based evaluation of variants

Among the 252 *BRCA2* missense variants with functional data, 186 had previously been observed by the collaborating clinical diagnostic laboratory (Ambry Genetics, Aliso Viejo, CA) and had undergone systematic review as recommended by the ACMG/AMP and the Clinical Genome Resource (ClinGen). In brief, the ACMG/AMP baseline recommendations combine evidence from population, computational, segregation, and functional data into a weighted scheme by applying supporting, moderate, strong, very strong, or stand-alone weights toward a final variant classification of benign (B), likely benign (LB), variant of uncertain significance (VUS), likely pathogenic (LP), or pathogenic (P).[6,11] An integrated approach based on the ACMG/AMP framework was previously used by this clinical laboratory for *BRCA2* variant classification.[11] The elements of clinical and pathological data used by Ambry Genetics for clinical evaluation of the variants presented in the current study based on a tailored ACMG/AMP model were available for integration with the functional assay data. The study was approved by Mayo Clinic institutional review board and the anlysis of the clinical-testing cohort was deemed exempt from review by the Western Institutional Review Board. Modifications to the ACMG/AMP framework used in this study are detailed in the following sections.

In silico *data (PP3/BP4)*

The meta-predictor BayesDel was used for *in silico* interpretation of the variants employing internal, gene-specific cutoffs as previously described.[21] Variants scoring <0.0560 were considered *in silico* tolerated (BP4), while variants scoring >0.431 were considered *in silico* deleterious (PP3). Variants between these scores were considered *in silico* inconclusive.

General population frequency data (BA1/BS1/PM2)

Empirically derived, general population (gnomAD) allele frequency thresholds of 0.1% for the BA1 (population-based allele frequency for a stand-alone benign classification) and 0.01% for the BS1 (allele frequency for a benign variant greater than expected for the related disorder) rules were applied. In addition, the PM2 rule for rare variants was applied only as a supporting line of evidence (PM2_supporting) because many benign variants are also rare in the population. For the purposes of this work, PM2_supporting was applied for variants with <0.001% total general population frequency, which equates to ≤1 heterozygote at well-covered positions with a maximum possible allele count of 282,912 in gnomAD v2.1.1.[22]

Well-studied functional domain (PM1)

PM1 is derived from a computational structural analysis based on protein modeling that incorporates secondary, tertiary, and quaternary structures, protein stability, known clinically important functional domains and motifs, and comparisons to known benign and pathogenic variants within the structure. Results from integrated structural analyses are applied as either PM1 or PM1_supporting depending on the amount of data available to inform the analysis.[11]

ClinVar mining

In order to understand the potential impact of functional data on the classification of variants in the public domain, we analyzed ClinVar data to compare the number of conflicting or VUS assertions for variants within the DBD and how these might be resolved with the uniform application of high-quality protein functional data. Only information from the ClinGen list of clinical laboratories meeting minimum requirements for data sharing to support quality assurance was used. ClinVar variant entries for which the collaborating clinical laboratory was the only submitter or the sole classification outlier were excluded. Final assertions for variants were categorized as “conflicting” or “non-conflicting” based on the ClinVar designation. Conflicting interpretations for variants from this study compared to ClinVar were further binned as either likely pathogenic/pathogenic (conflicting-LP/P) or likely benign/benign (conflicting-LB/B) based on the non-VUS classification.

Results

Functional assessment of missense variants by HDR assay

HDR assay results are provided for 53 variants, along with previously reported data for 199 variants, for a total of 252 missense variants (Figure 1; Table 1; Table S1).[9,10] Among the 252 variants, 90 were considered non-functional with HDR fold change <1.66, based on the upper 95% confidence interval, which is associated with probabilities of pathogenicity >0.99 (Table 1, Figure 1, Table S1). In addition, 162 variants were considered functional with HDR > 2.25, based on the lower 95% confidence interval, which is the threshold for probabilities of neutrality >0.95 (Table S1, Supplemental references). Non-functional variants ranged spatially from p.Gly2508Arg (c.7522G>C) to p.Ley3180Pro (c.9539T>C) and were evenly distributed in the Helical, OB1, and OB3 globular domains of the DBD. However, only seven variants in OB2 region (residues 2804–3054) and none of the 23 variants in the OB2-associated tower domain of BRCA2 (residues 2838–2962) had non-functional HDR effects (Figure 1). Several amino acids were found to be critical for function and sensitive to substitution. For instance, variants that changed residue 2619 from Trp to Gly, Ser, or Cys all resulted in loss of function. Similarly, variants that change residue 2723 from Asp to Asn, His, Tyr, Ala, Gly, and Val also consistently resulted in loss of function, suggesting that a negatively charged amino acid is required at this position. In contrast, differential effects on function were observed for two alterations in residue Leu3180, where a Pro substitution resulted in loss of function but an Arg substitution resulted in a functional protein (Figure 1, Table S1). Differential impact of substitutions on the same residue was also observed for p.Gly2508Ser/Arg (c.7522G>A/C), p.Ala2603Ser/Pro (c.7807G>T/C), p.Arg2625Lys/Ile (c.7874G>A/T), p.Ile2627Val/Phe/Asn (c.7879A>G/T, c.7880T>A), and p.Asn3124His/Ile (c.9370A>C, c.9371A>T), suggesting that the PM5 rule, dealing with a missense variant occurring at the same amino acid as another pathogenic missense variant, cannot be applied in an uninformed manner (Figure 1, Table S1).

Application of the HDR assay to the ACMG/AMP protein functional data (PS3/BS3) rule

The HDR assay qualifies as a standardized gold-standard assay on the basis of the updated guidance provided by the ClinGen Sequence Variant Interpretation (SVI) Working Group.[23] Specifically, (1) the V-C8 *brca2* deficient cell line is a physiologically relevant model system because it recapitulates many features that are consistent with human *BRCA2* deficiency, including chromosomal instability, abnormal centrosomes, reduced nuclear localization of RAD51, and sensitivity to DNA cross-linking agents;[8,24] (2) the data produced by the HDR assay are highly reproducible, and transient cDNA-based expression is carefully controlled by monitoring protein expression by immunoblot;[8–10] (3) this assay demonstrates sufficient dynamic range between functionally abnormal and functionally normal; and (4) this assay is calibrated on the basis of a set of 20 known pathogenic and 46 known benign missense variants that were classified with a multifactorial likelihood model and/or an ACMG/AMP-based model that excludes functional data.[11,14–16] The ClinGen SVI recommendations for applying weight to functional studies suggest an odds of pathogenicity (oddspath) > 18.7 for the application of both the PS3 and BS3 rules as strong lines of evidence.[12,23] On the basis of 20 pathogenic and 46 benign standards, the oddspath was calculated as 46.0, and when restricting to 10 pathogenic and 32 benign standards not used for the original identification of HDR thresholds, the oddspath was estimated at 32.0. Thus, strong evidence of pathogenicity (PS3) was applied to any variant with upper 99% confidence interval HDR score < 1.66 and strong evidence for a benign variant (BS3) was applied to any variant with lower 95% confidence interval HDR score > 2.25 (Figure 1; Table 1; Table S1). Because this assay cannot assess possible effects on RNA splicing, splice predictions and splicing data must be considered before classifying a variant as LB or B based on the HDR score. Thus, variants ascribed benign HDR functional weight (BS3) were classified as LB/B only if there was no predicted impact on splicing per the *in silico* program SpliceAI.[17] Of note, in the original ACMG/AMP guidelines from 2015, strong benign functional evidence (BS3), as a categorical line of evidence, was not sufficient to classify a variant as LB.[6] However, ClinGen SVI now recognizes BS3 as sufficient evidence for LB.[12]

Effect of protein functional data on ACMG/AMP-based variant classification

Among the 186 variants observed by the collaborating clinical laboratory, 154 (83%) were classified as VUSs in the absence of protein functional data. After applying HDR assay protein functional data, 86% (132/154) of these VUSs were reclassified as either LP/P (39/132) (48 after HDR versus 9 before HDR) or LB/B (93/132) (23 before HDR versus 116 after HDR) (Figure 2, Figure 3, Table S1). These variants had previously remained VUSs primarily because of rarity, which limited additional data from other lines of evidence in the ACMG/AMP-like classification framework (Table S1). The 22 variants that remained as VUSs after the application of functional data had either limited or conflicting data on the basis of ACMG/AMP classification guidelines (Table S1).

Concordance with other BRCA2 functional studies

The HDR functional dataset was completely concordant with three other functional studies that evaluated the ability for a human *BRCA2* variant to restore survival of *Brca2* null mouse embryonic stem cells (Table S1).[25–27] In another functional study, BRCA2 variants were evaluated for influence on sensitivity to PARP inhibitors in a *BRCA2*-deficient

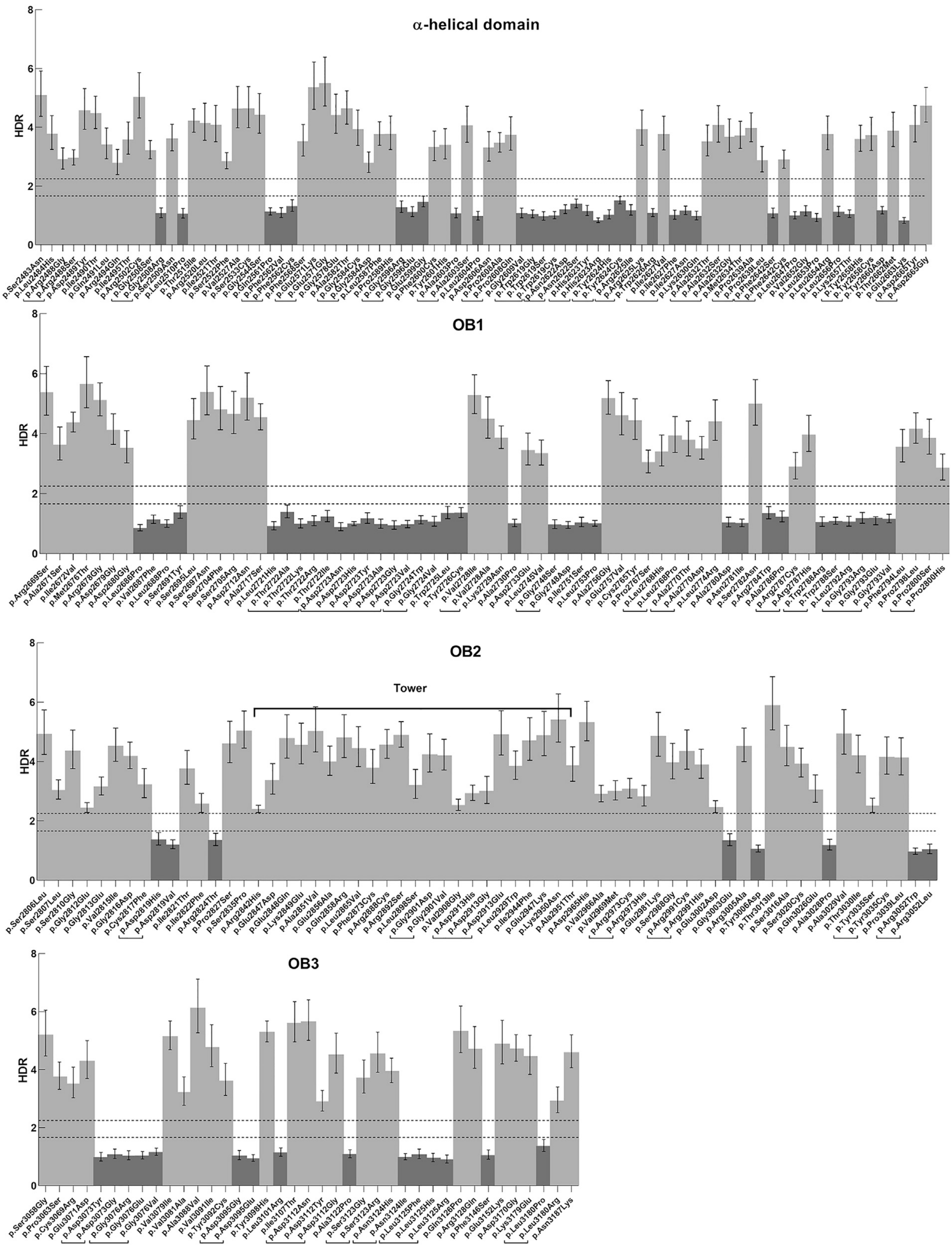

Figure 1. Homology-directed repair (HDR) activity
HDR activity of 252 *BRCA2* DNA-binding-domain (DBD) missense variants based on an HDR DR-GFP assay. The HDR fold-change based on proportions of GFP positive cells resulting from HDR activity is displayed on a linear scale between 1 (non-functional, p.Asp2723His) and 5 (functional, wild-type). Functional variants are shown in light gray, and non-functional variants are shown in dark gray. The 95% confidence intervals (CIs) for the HDR scores are included as a measure of the reproducibility of the HDR assay for each variant. Horizontal dotted lines represent 99% probability of pathogenicity (fold increase in GFP [+] cells < 1.66) and 95% probability of neutrality (fold increase in GFP [+] cells > 2.25). BRCA2 DBDs are denoted above each section, with the tower domain in OB2 indicated by a bracket. Different amino acid substitutions at the same position are grouped by brackets.

Table 1. BRCA2 variants with non-functional HDR score

Variant	HDR score (upper 95% CI)	ACMG/AMP codes: PS3/BS3[a] (function)	PM1 (structure)	PM5[b] (same residue)	PP1 (coseg)	PM3[c] FA/BP2 (healthy biallelic patients)	PP3/BP4[d,e] (BayesDel)	Frequency codes[f] (BA1/BS1/ PM2_Supp)	Classification data: Before class	Final class
p.Ala2603Pro	1.07 (1.25)	PS3	–	–	–	–	–	–	N/O	N/O
p.Ala2730Pro	1.01 (1.15)	PS3	PM1_supp	–	PP1	–	inc	–	VUS	LP
p.Ala2780Asp	1.04 (1.21)	PS3	–	–	–	–	inc	PM2_supp	VUS	VUS
p.Ala2786Pro	1.22 (1.43)	PS3	–	–	–	–	–	–	N/O	N/O
p.Ala3028Pro	1.18 (1.38)	PS3	PM1	–	PP1_mod	PM3	BP4	PM2_supp	LP	P
p.Ala3122Pro	1.09 (1.24)	PS3	–	–	–	–	–	–	N/O	N/O
p.Asp2723Ala	0.99 (1.15)	PS3	PM1	PM5	PP1	–	PP3	PM2_supp	LP	P
p.Asp2723Gly	0.94 (1.10)	PS3	–	PM5	–	–	PP3	PM2_supp	VUS	P
p.Asp2723His	1.00 (1.07)	PS3	–	–	–	–	PP3	PM2_supp	VUS	P
p.Asp2723Asn	0.89 (1.04)	PS3	PM1	–	–	–	inc	PM2_supp	VUS	LP
p.Asp2723Val	0.98 (1.11)	PS3	PM1	PM5	PP1	–	PP3	PM2_supp	LP	P
p.Asp2723Tyr	1.17 (1.36)	PS3	–	–	–	–	–	–	N/O	N/O
p.Asp2819His	1.37 (1.60)	PS3	–	–	–	–	–	–	N/O	N/O
p.Asp2819Val	1.20 (1.36)	PS3	–	–	–	–	inc	PM2_supp	VUS	VUS
p.Asp3073Gly	1.08 (1.26)	PS3	–	–	–	–	inc	PM2_supp	VUS	VUS
p.Asp3073Tyr	0.98 (1.15)	PS3	–	–	–	–	inc	PM2_supp	VUS	VUS
p.Asp3095Glu	0.95 (1.07)	PS3	PM1	–	–	–	inc	PM2_supp	VUS	P
p.Asp3095Gly	1.04 (1.21)	PS3	–	PM5	PP1	–	inc	PM2_supp	VUS	P
p.Glu2599Gly	1.46 (1.66)	PS3	–	–	–	–	inc	PM2_supp	VUS	VUS
p.Glu2663Lys	0.83 (0.93)	PS3	PM1	–	PP1	–	inc	PM2_supp	VUS	P
p.Phe2562Cys	1.32 (1.54)	PS3	–	–	–	–	inc	PM2_supp	VUS	VUS
p.Phe2562Val	1.08 (1.26)	PS3	–	–	–	–	–	–	N/O	N/O
p.Phe2642Ser	1.07 (1.25)	PS3	–	–	–	–	–	–	N/O	N/O
p.Phe3146Ser	1.06 (1.23)	PS3	–	–	–	–	inc	PM2_supp	VUS	VUS
p.Gly2508Arg	1.08 (1.26)	PS3	–	–	–	–	–	–	N/O	N/O
p.Gly2596Glu	1.12 (1.30)	PS3	–	–	–	–	PP3	PM2_supp	VUS	LP
p.Gly2596Arg	1.28 (1.49)	PS3	–	–	–	–	PP3	PM2_supp	VUS	LP
p.Gly2609Val	1.07 (1.25)	PS3	PM1_supp	–	–	–	inc	PM2_supp	VUS	LP
p.Gly2724Val	1.07 (1.24)	PS3	–	–	–	–	PP3	PM2_supp	VUS	LP
p.Gly2724Trp	1.12 (1.27)	PS3	–	–	–	–	–	–	N/O	N/O
p.Gly2748Asp	0.95 (1.08)	PS3	–	–	PP1	–	PP3	–	VUS	LP
p.Gly2748Ser	0.97 (1.13)	PS3	–	–	–	–	–	–	N/O	N/O
p.Gly2793Glu	1.19 (1.23)	PS3	PM1	–	–	–	PP3	PM2_supp	VUS	LP
p.Gly2793Arg	1.18 (1.37)	PS3	PM1_supp	PM5	–	–	PP3	–	VUS	P
p.Gly2793Val	1.16 (1.32)	PS3	–	PM5	–	–	PP3	PM2_supp	VUS	P
p.Gly3003Glu	1.35 (1.57)	PS3	–	–	–	–	inc	PM2_supp	VUS	VUS
p.Gly3076Glu	1.04 (1.18)	PS3	PM1	–	–	–	PP3	PM2_supp	VUS	P
p.Gly3076Arg	1.03 (1.21)	PS3	–	–	–	–	–	–	N/O	N/O

(*Continued on next page*)

Table 1. *Continued*

Variant	HDR score (upper 95% CI)	ACMG/AMP codes							Classification data	
		PS3/BS3[a] (function)	PM1 (structure)	PM5[b] (same residue)	PP1 (coseg)	PM3[c] FA/BP2 (healthy biallelic patients)	PP3/BP4[d,e] (BayesDel)	Frequency codes[f] (BA1/BS1/ PM2_Supp)	Before class	Final class
p.Gly3076Val	1.16 (1.30)	PS3	PM1	PM5	–	–	inc	PM2_supp	LP	P
p.His2623Arg	0.83 (0.92)	PS3	PM1	–	PP1	–	PP3	PM2_supp	LP	P
p.His2623Tyr	1.15 (1.35)	PS3	–	PM5	–	–	inc	PM2_supp	VUS	LP
p.Ile2627Phe	1.01 (1.18)	PS3	–	–	–	–	inc	PM2_supp	VUS	LP
p.Ile2627Asn	1.16 (1.32)	PS3	–	PM5	–	–	inc	PM2_supp	VUS	LP
p.Ile2751Ser	1.04 (1.21)	PS3	–	–	–	–	inc	PM2_supp	VUS	VUS
p.Lys2630Gln	0.98 (1.15)	PS3	–	–	–	–	–	–	N/O	N/O
p.Lys2657Thr	1.05 (1.19)	PS3	–	–	–	–	inc	PM2_supp	VUS	VUS
p.Leu2510Pro	1.06 (1.24)	PS3	PM1_supp	–	–	PM3_st	PP3	PM2_supp	LP	P
p.Leu2604Pro	0.98 (1.14)	PS3	PM1	–	–	–	PP3	PM2_supp	VUS	P
p.Leu2647Pro	1.00 (1.13)	PS3	–	–	PP1_mod	–	PP3	PM2_supp	VUS	P
p.Leu2653Pro	0.92 (1.07)	PS3	–	–	–	–	inc	PM2_supp	VUS	LP
p.Leu2656Pro	1.12 (1.31)	PS3	–	–	–	–	–	–	N/O	N/O
p.Leu2686Pro	0.86 (0.97)	PS3	PM1	–	–	PM3_st	inc	PM2_supp	LP	P
p.Leu2688Pro	1.00 (1.13)	PS3	–	–	PP1_mod	–	PP3	PM2_supp	VUS	P
p.Leu2721His	0.92 (1.07)	PS3	PM1	–	–	–	inc	PM2_supp	VUS	LP
p.Leu2753Pro	1.01 (1.11)	PS3	–	–	–	–	BP4	PM2_supp	VUS	VUS
p.Leu2792Arg	1.07 (1.24)	PS3	–	–	–	–	PP3	PM2_supp	VUS	LP
p.Leu3101Arg	1.15 (1.30)	PS3	PM1	–	PP1	PM3_st	inc	PM2_supp	P	P
p.Leu3125Phe	1.08 (1.26)	PS3	–	–	–	–	–	–	N/O	N/O
p.Leu3125His	0.96 (1.12)	PS3	–	–	–	–	–	–	N/O	N/O
p.Leu3125Arg	0.91 (1.06)	PS3	–	–	–	–	–	–	N/O	N/O
p.Leu3180Pro	1.37 (1.59)	PS3	–	–	–	–	–	–	N/O	N/O
p.Asn2622Asp	1.20 (1.36)	PS3	–	–	–	–	–	–	N/O	N/O
p.Asn2622Ser	1.40 (1.56)	PS3	–	–	–	–	inc	BS1 (.020% AFR)	VUS	VUS
p.Asn2781Ile	1.02 (1.15)	PS3	PM1_supp	–	–	–	inc	PM2_supp	VUS	LP
p.Asn3124Ile	0.99 (1.11)	PS3	PM1	–	–	–	inc	PM2_supp	VUS	P
p.Gln2561Pro	1.13 (1.26)	PS3	–	–	–	–	–	–	N/O	N/O
p.Arg2625Ile	1.17 (1.37)	PS3	–	–	–	–	–	–	N/O	N/O
p.Arg2784Trp	1.35 (1.57)	PS3	PM1_supp	–	PP1	PM3	inc	–	VUS	P
p.Arg2824Thr	1.36 (1.58)	PS3	–	–	–	–	–	–	N/O	N/O
p.Arg3052Leu	1.04 (1.21)	PS3	–	PM5	–	–	inc	PM2_supp	VUS	LP
p.Arg3052Trp	0.97 (1.08)	PS3	–	–	–	–	inc	–	VUS	P
p.Ser2691Tyr	1.37 (1.60)	PS3	–	–	–	–	–	–	N/O	N/O
p.Thr2722Ala	1.39 (1.62)	PS3	–	–	–	–	inc	PM2_supp	VUS	VUS
p.Thr2722Ile	1.24 (1.44)	PS3	–	–	–	–	inc	PM2_supp	VUS	VUS
p.Thr2722Lys	1.00 (1.16)	PS3	PM1	PM5	–	–	inc	PM2_supp	LP	P
p.Thr2722Arg	1.08 (1.26)	PS3	PM1	–	–	–	inc	PM2_supp	VUS	P

(*Continued on next page*)

Table 1. *Continued*

Variant	HDR score (upper 95% CI)	ACMG/AMP codes PS3/BS3[a] (function)	PM1 (structure)	PM5[b] (same residue)	PP1 (coseg)	PM3[c] FA/BP2 (healthy biallelic patients)	PP3/BP4[d, e] (BayesDel)	Frequency codes[f] (BA1/BS1/ PM2_Supp)	Classification data Before class	Final class
p.Val2652Gly	1.14 (1.33)	PS3	–	–	–	–	–	–	N/O	N/O
p.Val2687Phe	1.14 (1.29)	PS3	PM1	–	–	–	inc	PM2_supp	VUS	LP
p.Trp2619Cys	1.00 (1.14)	PS3	–	–	–	–	PP3	PM2_supp	VUS	LP
p.Trp2619Gly	1.04 (1.18)	PS3	PM1	–	–	–	PP3	PM2_supp	VUS	P
p.Trp2619Ser	0.97 (1.13)	PS3	–	–	–	–	–	–	N/O	N/O
p.Trp2626Arg	1.09 (1.23)	PS3	PM1	–	–	–	PP3	PM2_supp	VUS	P
p.Trp2725Leu	1.35 (1.58)	PS3	–	–	–	–	–	–	N/O	N/O
p.Trp2788Arg	1.05 (1.23)	PS3	PM1_supp	–	–	–	inc	PM2_supp	VUS	LP
p.Trp2788Ser	1.09 (1.21)	PS3	–	PM5	–	–	inc	PM2_supp	VUS	LP
p.Tyr2624Cys	1.51 (1.64)	PS3	PM1_supp	–	–	–	PP3	PM2_supp	VUS	LP
p.Tyr2624His	1.02 (1.19)	PS3	–	–	–	–	–	–	N/O	N/O
p.Tyr2660Asp	1.17 (1.30)	PS3	–	–	–	–	–	–	N/O	N/O
p.Tyr2726Cys	1.36 (1.54)	PS3	PM1	–	–	–	PP3	PM2_supp	VUS	P
p.Tyr3006Asp	1.06 (1.18)	PS3	–	–	–	–	inc	PM2_supp	VUS	VUS

Abbreviations are as follows: CI, 95% confidence interval; coseg, cosegregation; FA, Fanconi anemia; class, variant classification; N/A, not applicable; st, strong; mod, moderate; supp, supporting; N/O, not observed; LP, likely pathogenic; P, pathogenic; LB, likely benign; B, benign; VUS, variant of uncertain significance; N/D, no deposit; inc, inconclusive; AFR, African American.
[a]PS3 is applied only when the upper 95% CI is <1.66.
[b]PM5 (same residue as known pathogenic variant) use is dependent on additional predictive data indicating the variant in question will be worse than the initial pathogenic missense variant at the amino acid position (e.g., Grantham score or fold energy change).
[c]Confirmed phenotype, confirmed trans (PM3_strong per each); confirmed phenotype, presumed trans (PM3 per each); consistent phenotype, confirmed trans (PM3 per each). See Table S1 for citations.
[d]BayesDel BRCA2-specific thresholds established (see citation) and modified on the basis of continued internal calibrations: <0.0560 is tolerated; >0.4310 (deleterious); non-SNV variants were ascertained with PROVEAN, and those scoring below the internal threshold of −6 were considered deleterious.
[e]BP4 *in silico* is upgraded to BP4_strong on the basis of a stringent internal threshold for conservation based on an extensive multiple sequence alignment.
[f]Proposed cutoffs for *BRCA2* for BA1 and BS1 are 0.1% and 0.01%, respectively. Application of these codes is based on filtering allele frequency (FAF) in gnomAD from January 2020–whichever was lower between genomes and exomes; PM2_supporting is based on the presence of ≤1 carrier in the gnomAD total population (accessed January 2020).

cell line.[28] Among the 63 variants that overlapped with the current HDR functional study, all variants that were non-functional by HDR also demonstrated sensitivity to one or more drugs (non-functional-fClass 4 or fClass 5) or were inconclusive (fClass 3). None were considered resistant (functional-fClass 1 or fClass 2) for any of the four drugs in the sensitivity assays. However, among the variants that were functional by HDR assay, six had non-functional scores (fClass 4 or fClass 5) for at least one drug in the drug sensitivity assay (Table S1, red text). Cells carrying variants p.Val2908Gly (c.8723T>G) and p.Val2969Met (c.8905G>A) were consistently sensitive (non-functional) across all four drugs, but both of these variants have non-conflicting LB assertions in ClinVar on the basis of a multifactorial score influenced by both family history and co-segregation scores, suggesting that further calibration of the drug sensitivity assay is needed.

Concordance with ClinVar classification of variants

To determine the potential impact of high-quality functional data on variant classification in the public domain, we reviewed ClinVar submissions for missense variants in the BRCA2 DBD on January 31, 2020. Among the 987 missense variants in the DBD, 54 were excluded as a result of having no submissions from laboratories meeting minimum requirements for data sharing. A further 169 variants were excluded because the collaborating clinical laboratory provided the sole assertions, was the sole contributor meeting minimum requirements for data sharing, or was the sole classification outlier among the contributors meeting minimum requirements for data sharing. The remaining 764 variants represent ClinVar submissions from at least one contributing laboratory that met minimum requirements for data sharing. Importantly, 93% (709/764) of these variants were classified as VUSs or had unresolved conflicting classifications in ClinVar.

Among the 186 functionally assessed variants in this study that were also observed by Ambry Genetics, 171 had ClinVar assertions that were not dependent on this clinical laboratory (Table S1, Figure 4). Among these, 36 had an LP/P final classification (including functional

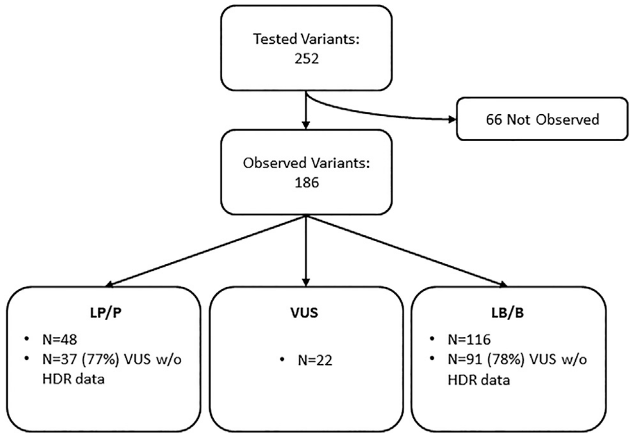

Figure 2. Variant workflow
Summary of the number of variants subjected to the HDR assay and to variant interpretation. The number of variants within each classification outcome (LP/P, VUS, and LB/B) (first bullet, lower panels) and classification rates for VUS in the absence of protein functional data (second bullet, lower panels) are shown. P, pathogenic; LP, likely pathogenic; VUS, variant of uncertain significance; LB, likely benign; B, benign.

data) in this study, of which 39% (14/36) had no-conflict LP/P ClinVar assertions, 30.5% (11/36) had no-conflict VUS ClinVar assertions, and 30.5% (11/36) had conflicting LP/P ClinVar assertions. None of the variants with a final classification of LP/P variants had opposing LB/B assertions in ClinVar (Table S1, Figure 4). Conversely, 99 variants with non-excluded ClinVar assertions had an LB/B final classification. Of these, 22% (22/99) had a no-conflict LB/B ClinVar assertion, 45% (45/99) had no-conflict VUS ClinVar assertions, and 32% (32/99) had conflicting LB/B ClinVar assertions. No internally classified LB/B variants had opposing LP/P assertions (Table S1; Figure 4). The resolution of 132 VUSs by the incorporation of high-quality functional data impacted 1,900 individuals tested at the collaborating clinical laboratory (Figure 3).

Discussion

This study describes the application of a large clinical, pathology, and genetic dataset along with protein functional codes weighted according to ClinGen SVI guidelines for the classification of *BRCA2* variants with ACMG/AMP guidelines. Several other studies of missense VUSs in BRCA2 based on multifactorial likelihood classification models incorporating personal and family history of cancer, co-segregation of variants with disease, and *in silico* sequence-based prediction models have been published.[14–16] These quantitative models, that do not incorporate protein functional data for variant classification are useful for comparisons with results from the ACMG/AMP rules-based approach reported here. Among 49 variants that were commonly evaluated by HDR assay and by quantitative classification models in other studies, concordance was observed for 48 variants (98%) (Table S1).[14–16] The sole outlier in this comparison was BRCA2 p.Arg2502Cys (c.7504C>T). This variant was classified as LB/B in ClinVar by multiple submitters, and was functional in the HDR assay, but remained as an unclassified VUS in a recent multifactorial analysis.[16] Importantly, the overwhelming concordance of HDR functional data with these prior studies, as well as other variants that achieved an LP/P or LB/B classification without the use of functional data, allows for the stringent validation and weighting of this functional assay as a strong line of evidence in an ACMG/AMP-based classification scheme.[14–16]

Of note, among the 22 variants that remained a VUS for the collaborating clinical laboratory, 13 had limited data from sources other than the functional assay for classification purposes and none of these 13 had resolved ClinVar assertions. Of the variants that remained a VUS after the application of functional weight, eight had conflicting data and two of these eight variants had conflicting-LB/B assertions in ClinVar. Reasons for conflict include *in silico* BayesDel prediction scores (p.Asp2679Gly (c.8036A>G), p.Gly2812Glu (c.8435G>A), p.Gly2813Glu (c.8438G>A), and p.Leu2753Pro (c.8258T>C)), general population frequency (p.Asn2622Ser (c.7865A>G)), or a line of evidence based on structure or where other variants in the same amino acids were known to be pathogenic or benign (p.Ile2627Val, p.Asn3124His, and p.Tyr2658His [c.7972T>C]).

Interestingly, over 53% (99/186) of the variants observed by the collaborating clinical laboratory had inconclusive scores with the BayesDel *in silico* method based on stringent internally calibrated thresholds for *BRCA2* (Table S1).[21] The inconclusive scores did not favor functional or non-functional variants but were spread relatively evenly across the HDR results, suggesting poor sensitivity for the BayesDel method relative to the functional data.[10] These data

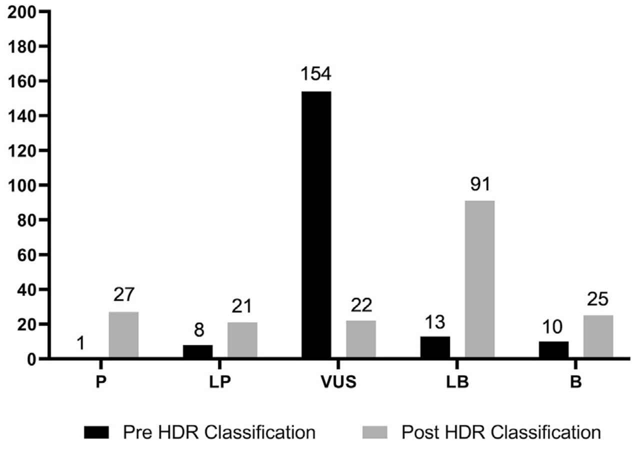

Figure 3. Influence of HDR functional data on variant classification with an ACMG/AMP-like model
Classification of variants before (black bars) and after (gray bars) the application of protein functional data. The number of variants is indicated above the bar. P, pathogenic; LP, likely pathogenic; VUS, variant of uncertain significance; LB, likely benign; B, benign.

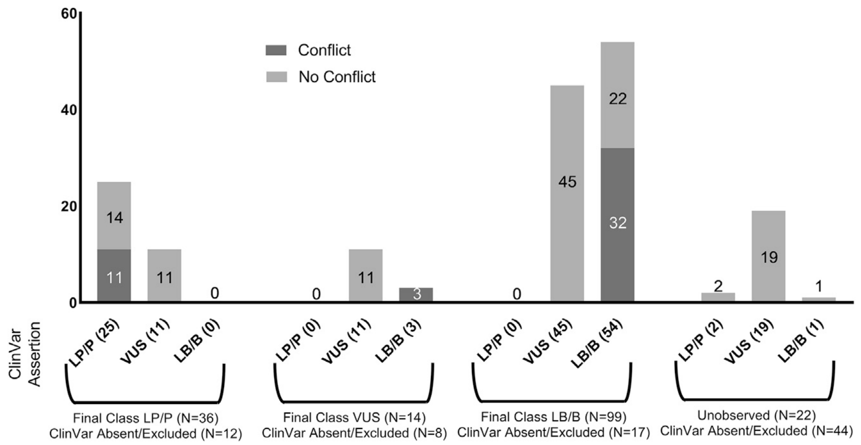

Figure 4. Final variant classification compared to ClinVar assertions
Variants are grouped according to final classification (LP/P, VUS, LB/B, and unobserved) and further subdivided into the general assertion provided by ClinVar (LP/P, VUS, and LB/B). Each bar is shaded to indicate a conflicting ClinVar assertion (dark gray) or a non-conflicting assertion (light gray). Categories without a conflicting or non-conflicting sub-category (VUS) are shown as "0." The total number of variants in each final classification category is represented in parentheses, and the number of variants designated as conflicting or non-conflicting is indicated within the bar. P, pathogenic; LP, likely pathogenic; VUS, variant of uncertain significance; LB, likely benign; B, benign.

highlight the need for better gene-specific or even domain-specific *in silico* predictors for the BRCA2 DBD, such as the BRCA-ML ensemble model.[29] The combination of a high-quality *in silico* predictor with high-quality functional data to supplement clinical and genetic data is expected to result in the classification of many rare variants in the DBD of BRCA2.

In comparing the HDR functional data and final classifications with non-excluded ClinVar assertions, the majority of variants with previously reported HDR functional data (84/117 = 72%) had unresolved ClinVar assertions of VUSs or conflicting. This suggests that functional data are inconsistently applied in classification schema among ClinVar submitters. The results provided in this study may lead to a more uniform application of a strong line of functional evidence for the BRCA2 HDR assay among all ACMG/AMP-based classification schema for *BRCA2* variants.

This study had a high success rate, with 86% (132/154) of observed VUSs classified as LP/P or LB/B because of the application of a strong line of evidence for high-quality protein functional data provided by the BRCA2 HDR functional assay. Assuming a similar resolution rate, an additional 657 of 764 identified missense variants in the BRCA2 DBD with unresolved VUSs or conflicting ClinVar assertions may achieve a classification of LB/B or LP/P through the addition of HDR functional data. These data highlight the substantial impact that high-quality functional data can have on the classification of rare variants with the ACMG/AMP guidelines. The finding that the results of the current study impacted genetic diagnoses of 1,900 probands also highlights the substantial impact that high-quality functional data can have on individuals undergoing hereditary cancer genetic testing. Further incorporation of functional assay data into VUS classification models is expected to result in substantially reduced *BRCA2* VUS rates and impact the clinical management of many tested probands and family members.

A limitation of the approach is that the specific assay is only validated for variants in the BRCA2 DBD. However, as no missense variants from other domains have been classified as pathogenic, it remains possible that only missense variants in the DBD increase risk of cancer. The possibility also exists that BRCA2 may have multiple functions associated with cancer risk, although evidence to date shows that all pathogenic missense variants influence HDR activity. Furthermore, as shown in this study, additional classification criteria in combination with functional assay results may always be needed for LP/P classification of VUSs.

Data and code availability

The published article includes all datasets generated or analyzed during this study.

Supplemental Information

Supplemental Data can be found online at https://doi.org/10.1016/j.ajhg.2021.02.005.

Acknowledgments

The study was funded by National Institute of Health, National Cancer Institute grants (R35CA253187, R01CA192393, R01CA116167, and R01CA225662); the Specialized Program of Research Excellence (SPORE) in Breast Cancer (P50CA116201) to Mayo Clinic; and the Breast Cancer Research Foundation.

Declaration of interests

K.F., K.M.D., A.M.D., H.L., M.E.R., E.C., and T.P. are employees of Ambry Genetics. F.J.C. has received consulting fees from Astrazeneca. The remaining authors declare no competing interests.

Received: November 3, 2020
Accepted: February 2, 2021
Published: February 19, 2021

Web resources

Clinical laboratories meeting minimum requirements for data sharing to support quality assurance, https://www.clinicalgenome.org/tools/clinical-lab-data-sharing-list/

GenBank, https://www.ncbi.nlm.nih.gov/genbank/

NCCN clinical practice guidelines in oncology for breast cancer, https://www.nccn.org/professionals/physician_gls/pdf/breast.pdf

NCCN clinical practice guidelines in oncology for ovarian cancer, https://www.nccn.org/professionals/physician_gls/pdf/ovarian.pdf

Omim, https://www.omim.org/entry/600185

References

1. Kuchenbaecker, K.B., Hopper, J.L., Barnes, D.R., Phillips, K.A., Mooij, T.M., Roos-Blom, M.J., Jervis, S., van Leeuwen, F.E., Milne, R.L., Andrieu, N., et al.; BRCA1 and BRCA2 Cohort Consortium (2017). Risks of Breast, Ovarian, and Contralateral Breast Cancer for BRCA1 and BRCA2 Mutation Carriers. JAMA *317*, 2402–2416.
2. Kim, G., Ison, G., McKee, A.E., Zhang, H., Tang, S., Gwise, T., Sridhara, R., Lee, E., Tzou, A., Philip, R., et al. (2015). FDA Approval Summary: Olaparib Monotherapy in Patients with Deleterious Germline BRCA-Mutated Advanced Ovarian Cancer Treated with Three or More Lines of Chemotherapy. Clin. Cancer Res. *21*, 4257–4261.
3. Balasubramaniam, S., Beaver, J.A., Horton, S., Fernandes, L.L., Tang, S., Horne, H.N., Liu, J., Liu, C., Schrieber, S.J., Yu, J., et al. (2017). FDA Approval Summary: Rucaparib for the Treatment of Patients with Deleterious *BRCA* Mutation-Associated Advanced Ovarian Cancer. Clin. Cancer Res. *23*, 7165–7170.
4. Landrum, M.J., Lee, J.M., Benson, M., Brown, G., Chao, C., Chitipiralla, S., Gu, B., Hart, J., Hoffman, D., Hoover, J., et al. (2016). ClinVar: public archive of interpretations of clinically relevant variants. Nucleic Acids Res. *44* (D1), D862–D868.
5. Couch, F.J., Shimelis, H., Hu, C., Hart, S.N., Polley, E.C., Na, J., Hallberg, E., Moore, R., Thomas, A., Lilyquist, J., et al. (2017). Associations Between Cancer Predisposition Testing Panel Genes and Breast Cancer. JAMA Oncol. *3*, 1190–1196.
6. Richards, S., Aziz, N., Bale, S., Bick, D., Das, S., Gastier-Foster, J., Grody, W.W., Hegde, M., Lyon, E., Spector, E., et al. (2015). Standards and guidelines for the interpretation of sequence variants: a joint consensus recommendation of the American College of Medical Genetics and Genomics and the Association for Molecular Pathology. Genet. Med. *17*, 405–424.
7. Brnich, S.E., Rivera-Muñoz, E.A., and Berg, J.S. (2018). Quantifying the potential of functional evidence to reclassify variants of uncertain significance in the categorical and Bayesian interpretation frameworks. Hum. Mutat. *39*, 1531–1541.
8. Guidugli, L., Pankratz, V.S., Singh, N., Thompson, J., Erding, C.A., Engel, C., Schmutzler, R., Domchek, S., Nathanson, K., Radice, P., et al. (2013). A classification model for BRCA2 DNA binding domain missense variants based on homology-directed repair activity. Cancer Res. *73*, 265–275.
9. Guidugli, L., Shimelis, H., Masica, D.L., Pankratz, V.S., Lipton, G.B., Singh, N., Hu, C., Monteiro, A.N.A., Lindor, N.M., Goldgar, D.E., et al. (2018). Assessment of the Clinical Relevance of BRCA2 Missense Variants by Functional and Computational Approaches. Am. J. Hum. Genet. *102*, 233–248.
10. Hart, S.N., Hoskin, T., Shimelis, H., Moore, R.M., Feng, B., Thomas, A., Lindor, N.M., Polley, E.C., Goldgar, D.E., Iversen, E., et al. (2019). Comprehensive annotation of BRCA1 and BRCA2 missense variants by functionally validated sequence-based computational prediction models. Genet. Med. *21*, 71–80.
11. Pesaran, T., Karam, R., Huether, R., Li, S., Farber-Katz, S., Chamberlin, A., Chong, H., LaDuca, H., and Elliott, A. (2016). Beyond DNA: An Integrated and Functional Approach for Classifying Germline Variants in Breast Cancer Genes. Int. J. Breast Cancer *2016*, 2469523.
12. Tavtigian, S.V., Greenblatt, M.S., Harrison, S.M., Nussbaum, R.L., Prabhu, S.A., Boucher, K.M., Biesecker, L.G.; and ClinGen Sequence Variant Interpretation Working Group (2018). Modeling the ACMG/AMP variant classification guidelines as a Bayesian classification framework. Genet. Med. *20*, 1054–1060.
13. Goldgar, D.E., Easton, D.F., Deffenbaugh, A.M., Monteiro, A.N., Tavtigian, S.V., Couch, F.J.; and Breast Cancer Information Core (BIC) Steering Committee (2004). Integrated evaluation of DNA sequence variants of unknown clinical significance: application to BRCA1 and BRCA2. Am. J. Hum. Genet. *75*, 535–544.
14. Lindor, N.M., Guidugli, L., Wang, X., Vallée, M.P., Monteiro, A.N., Tavtigian, S., Goldgar, D.E., and Couch, F.J. (2012). A review of a multifactorial probability-based model for classification of BRCA1 and BRCA2 variants of uncertain significance (VUS). Hum. Mutat. *33*, 8–21.
15. Easton, D.F., Deffenbaugh, A.M., Pruss, D., Frye, C., Wenstrup, R.J., Allen-Brady, K., Tavtigian, S.V., Monteiro, A.N., Iversen, E.S., Couch, F.J., and Goldgar, D.E. (2007). A systematic genetic assessment of 1,433 sequence variants of unknown clinical significance in the BRCA1 and BRCA2 breast cancer-predisposition genes. Am. J. Hum. Genet. *81*, 873–883.
16. Parsons, M.T., Tudini, E., Li, H., Hahnen, E., Wappenschmidt, B., Feliubadaló, L., Aalfs, C.M., Agata, S., Aittomäki, K., Alducci, E., et al.; KConFab Investigators (2019). Large scale multifactorial likelihood quantitative analysis of BRCA1 and BRCA2 variants: An ENIGMA resource to support clinical variant classification. Hum. Mutat. *40*, 1557–1578.
17. Jaganathan, K., Kyriazopoulou Panagiotopoulou, S., McRae, J.F., Darbandi, S.F., Knowles, D., Li, Y.I., Kosmicki, J.A., Arbelaez, J., Cui, W., Schwartz, G.B., et al. (2019). Predicting Splicing from Primary Sequence with Deep Learning. Cell *176*, 535–548.e24.
18. Farrugia, D.J., Agarwal, M.K., Pankratz, V.S., Deffenbaugh, A.M., Pruss, D., Frye, C., Wadum, L., Johnson, K., Mentlick, J., Tavtigian, S.V., et al. (2008). Functional assays for classification of BRCA2 variants of uncertain significance. Cancer Res. *68*, 3523–3531.
19. Wu, K., Hinson, S.R., Ohashi, A., Farrugia, D., Wendt, P., Tavtigian, S.V., Deffenbaugh, A., Goldgar, D., and Couch, F.J. (2005). Functional evaluation and cancer risk assessment of BRCA2 unclassified variants. Cancer Res. *65*, 417–426.
20. Goodrich, B., Gabry, J., Ali, I., and Brilleman, S. (2018). rstanarm: Bayesian applied regression modeling via Stan. (R package version 2.17.4).
21. Tian, Y., Pesaran, T., Chamberlin, A., Fenwick, R.B., Li, S., Gau, C.L., Chao, E.C., Lu, H.M., Black, M.H., and Qian, D. (2019). REVEL and BayesDel outperform other in silico meta-predictors for clinical variant classification. Sci. Rep. *9*, 12752.
22. Church, D.M. (2020). Thousands of human sequences provide deep insight into single genomes. Nature *581*, 385–386.
23. Brnich, S.E., Abou Tayoun, A.N., Couch, F.J., Cutting, G.R., Greenblatt, M.S., Heinen, C.D., Kanavy, D.M., Luo, X.,

McNulty, S.M., Starita, L.M., et al.; Clinical Genome Resource Sequence Variant Interpretation Working Group (2019). Recommendations for application of the functional evidence PS3/BS3 criterion using the ACMG/AMP sequence variant interpretation framework. Genome Med. *12*, 3.

24. Kraakman-van der Zwet, M., Overkamp, W.J., van Lange, R.E., Essers, J., van Duijn-Goedhart, A., Wiggers, I., Swaminathan, S., van Buul, P.P., Errami, A., Tan, R.T., et al. (2002). Brca2 (XRCC11) deficiency results in radioresistant DNA synthesis and a higher frequency of spontaneous deletions. Mol. Cell. Biol. *22*, 669–679.

25. Biswas, K., Das, R., Eggington, J.M., Qiao, H., North, S.L., Stauffer, S., Burkett, S.S., Martin, B.K., Southon, E., Sizemore, S.C., et al. (2012). Functional evaluation of BRCA2 variants mapping to the PALB2-binding and C-terminal DNA-binding domains using a mouse ES cell-based assay. Hum. Mol. Genet. *21*, 3993–4006.

26. Kuznetsov, S.G., Liu, P., and Sharan, S.K. (2008). Mouse embryonic stem cell-based functional assay to evaluate mutations in BRCA2. Nat. Med. *14*, 875–881.

27. Mesman, R.L.S., Calleja, F., Hendriks, G., Morolli, B., Misovic, B., Devilee, P., van Asperen, C.J., Vrieling, H., and Vreeswijk, M.P.G. (2019). The functional impact of variants of uncertain significance in BRCA2. Genet. Med. *21*, 293–302.

28. Ikegami, M., Kohsaka, S., Ueno, T., Momozawa, Y., Inoue, S., Tamura, K., Shimomura, A., Hosoya, N., Kobayashi, H., Tanaka, S., and Mano, H. (2020). High-throughput functional evaluation of BRCA2 variants of unknown significance. Nat. Commun. *11*, 2573.

29. Hart, S.N., Polley, E.C., Shimelis, H., Yadav, S., and Couch, F.J. (2020). Prediction of the functional impact of missense variants in BRCA1 and BRCA2 with BRCA-ML. NPJ Breast Cancer *6*, 13.

The American Journal of Human Genetics, Volume *108*

Supplemental data

Strong functional data for pathogenicity or neutrality classify BRCA2 DNA-binding-domain variants of uncertain significance

Marcy E. Richardson, Chunling Hu, Kun Y. Lee, Holly LaDuca, Kelly Fulk, Kate M. Durda, Ashley M. Deckman, David E. Goldgar, Alvaro N.A. Monteiro, Rohan Gnanaolivu, Steven N. Hart, Eric C. Polley, Elizabeth Chao, Tina Pesaran, and Fergus J. Couch

Supplemental Data

Table S1- HDR functional assay results for all 252 variants. Results from other BRCA2 functional studies, multifactorial analyses, and splice predictions are shown to allow for comparison with HDR data.

Supplemental references

1. Guidugli, L., Pankratz, V.S., Singh, N., Thompson, J., Erding, C.A., Engel, C., Schmutzler, R., Domchek, S., Nathanson, K., Radice, P., et al. (2013). A classification model for BRCA2 DNA binding domain missense variants based on homology-directed repair activity. Cancer Res 73, 265-275.

2. Guidugli, L., Shimelis, H., Masica, D.L., Pankratz, V.S., Lipton, G.B., Singh, N., Hu, C., Monteiro, A.N.A., Lindor, N.M., Goldgar, D.E., et al. (2018). Assessment of the Clinical Relevance of BRCA2 Missense Variants by Functional and Computational Approaches. Am J Hum Genet 102, 233-248.

3. Hart, S.N., Hoskin, T., Shimelis, H., Moore, R.M., Feng, B., Thomas, A., Lindor, N.M., Polley, E.C., Goldgar, D.E., Iversen, E., et al. (2019). Comprehensive annotation of BRCA1 and BRCA2 missense variants by functionally validated sequence-based computational prediction models. Genetics in medicine : official journal of the American College of Medical Genetics 21, 71-80.

4. Mesman, R.L.S., Calleja, F., Hendriks, G., Morolli, B., Misovic, B., Devilee, P., van Asperen, C.J., Vrieling, H., and Vreeswijk, M.P.G. (2019). The functional impact of variants of uncertain significance in BRCA2. Genetics in medicine : official journal of the American College of Medical Genetics 21, 293-302.

5. Biswas, K., Das, R., Eggington, J.M., Qiao, H., North, S.L., Stauffer, S., Burkett, S.S., Martin, B.K., Southon, E., Sizemore, S.C., et al. (2012). Functional evaluation of BRCA2 variants mapping to

the PALB2-binding and C-terminal DNA-binding domains using a mouse ES cell-based assay. Hum Mol Genet 21, 3993-4006.

6. Kuznetsov, S.G., Liu, P., and Sharan, S.K. (2008). Mouse embryonic stem cell-based functional assay to evaluate mutations in BRCA2. Nat Med 14, 875-881.

7. Ikegami, M., Kohsaka, S., Ueno, T., Momozawa, Y., Inoue, S., Tamura, K., Shimomura, A., Hosoya, N., Kobayashi, H., Tanaka, S., et al. (2020). High-throughput functional evaluation of BRCA2 variants of unknown significance. Nat Commun 11, 2573.

8. Alter, B.P., Rosenberg, P.S., and Brody, L.C. (2007). Clinical and molecular features associated with biallelic mutations in FANCD1/BRCA2. J Med Genet 44, 1-9.

9. Dodgshun, A.J., Sexton-Oates, A., Saffery, R., and Sullivan, M.J. (2016). Biallelic FANCD1/BRCA2 mutations predisposing to glioblastoma multiforme with multiple oncogenic amplifications. Cancer Genet 209, 53-56.

10. Hirsch, B., Shimamura, A., Moreau, L., Baldinger, S., Hag-alshiekh, M., Bostrom, B., Sencer, S., and D'Andrea, A.D. (2004). Association of biallelic BRCA2/FANCD1 mutations with spontaneous chromosomal instability and solid tumors of childhood. Blood 103, 2554-2559.

11. Tian, Y., Pesaran, T., Chamberlin, A., Fenwick, R.B., Li, S., Gau, C.L., Chao, E.C., Lu, H.M., Black, M.H., and Qian, D. (2019). REVEL and BayesDel outperform other in silico meta-predictors for clinical variant classification. Sci Rep 9, 12752.

12. Lindor, N.M., Guidugli, L., Wang, X., Vallee, M.P., Monteiro, A.N., Tavtigian, S., Goldgar, D.E., and Couch, F.J. (2012). A review of a multifactorial probability-based model for classification of BRCA1 and BRCA2 variants of uncertain significance (VUS). Hum Mutat 33, 8-21.

13. Easton, D.F., Deffenbaugh, A.M., Pruss, D., Frye, C., Wenstrup, R.J., Allen-Brady, K., Tavtigian, S.V., Monteiro, A.N., Iversen, E.S., Couch, F.J., et al. (2007). A systematic genetic assessment of 1,433

sequence variants of unknown clinical significance in the BRCA1 and BRCA2 breast cancer-predisposition genes. Am J Hum Genet 81, 873-883.

14. Parsons, M.T., Tudini, E., Li, H., Hahnen, E., Wappenschmidt, B., Feliubadalo, L., Aalfs, C.M., Agata, S., Aittomaki, K., Alducci, E., et al. (2019). Large scale multifactorial likelihood quantitative analysis of BRCA1 and BRCA2 variants: An ENIGMA resource to support clinical variant classification. Hum Mutat 40, 1557-1578.
